# Psychosis in Laurence-Moon Syndrome: A Case Report

**DOI:** 10.7759/cureus.72003

**Published:** 2024-10-21

**Authors:** Nojoud Al Fareh, Marwah Abbas, Fahad D Alosaimi

**Affiliations:** 1 Department of Psychiatry, King Khalid University, Abha, SAU; 2 Psychosomatic Medicine Fellowship Program, Department of Psychiatry, King Saud Medical City, Riyadh, SAU; 3 Department of Psychiatry, King Saud University, Riyadh, SAU

**Keywords:** antipsychotic treatment, aripiprazole, auditory hallucinations, laurence-moon syndrome, multidisciplinary approach, obesity, psychotic disorder

## Abstract

Background: Laurence-Moon syndrome (LMS) is a rare autosomal recessive genetic disorder characterized by a combination of neurological, visual, and endocrine abnormalities. The coexistence of psychiatric disorders in LMS patients can complicate clinical management due to the intricate interplay between neurodevelopmental and psychiatric symptoms. A review of the literature failed to identify a similar case study published in major databases, including PubMed, highlighting the significance of this case report. Understanding these complexities is crucial for improving treatment strategies and patient outcomes.

Case presentation: A 35-year-old single Saudi male with LMS developed psychiatric symptoms, including social isolation and reduced family interaction since childhood, which worsened over the past two years as his vision deteriorated. The patient also exhibited elevated cholesterol levels and obesity, further complicating his treatment. Two years before seeking psychiatric help, he began experiencing severe auditory hallucinations, believing that his mother engaged in inappropriate activities, which led to increased fear, aggressive behavior, and neglect of basic needs. He was diagnosed with a psychotic disorder related to LMS and was initially treated with aripiprazole at a dose of 20 mg once daily, which significantly improved his symptoms but caused a resting tremor. When the dose was reduced to 10 mg daily, his psychotic symptoms returned, necessitating an increase to 15 mg daily, which stabilized his condition. A trial of escitalopram was ineffective and subsequently discontinued.

Conclusion: This case study provides valuable insights into the manifestations and management of psychosis in patients with LMS. A multidisciplinary approach involving psychiatry, neurology, endocrinology, and metabolic care is essential for comprehensive treatment. We recommend genetic testing and counseling for the patient’s family, particularly during family planning, with non-invasive prenatal testing (NIPT) for siblings after marriage. Further research is needed to develop targeted treatment strategies and explore the long-term outcomes of psychotropic medications in LMS, aiming to optimize treatment regimens and improve patient quality of life.

## Introduction

Laurence-Moon syndrome (LMS) is a rare autosomal recessive genetic multisystem disorder characterized by a combination of neurological, visual, and endocrine abnormalities [1,2]. It belongs to a group of ciliopathies-disorders caused by mutations in genes related to ciliary function. LMS is primarily associated with mutations in the *PNPLA6* gene, although other loci have also been implicated. These mutations disrupt ciliary function, leading to the diverse and multisystem manifestations observed in affected individuals, most of whom are children of consanguineous parents [3].

The primary features of LMS include cone-rod dystrophy, polydactyly, obesity, learning difficulties, hypogonadism in males, and renal anomalies. Secondary features consist of speech disorders, brachydactyly, developmental delay, polyuria/polydipsia, ataxia, poor coordination or clumsiness, diabetes mellitus, left ventricular hypertrophy, liver fibrosis, spasticity, and hearing loss. Additionally, short stature, dental crowding, hypermobile or lax joints, and early-onset osteoarthritis have also been reported [4].

LMS shares clinical features with other genetic disorders, such as Bardet-Biedl syndrome (BBS) and Oliver-McFarlane syndrome (OMS). Differentiating these syndromes is crucial for proper diagnosis and management, as they exhibit subtle but important clinical differences. For example, while both LMS and BBS present with retinal degeneration and obesity, LMS is more strongly associated with neurological impairments, such as spastic paraplegia, and lacks some of the renal manifestations commonly seen in BBS [5].

Psychiatric comorbidities in individuals with LMS are of particular concern due to the added layer of complexity they bring to the clinical management of the disorder [2,6]. Although the relationship between LMS and psychiatric disorders has not been extensively studied, emerging evidence suggests a possible comorbidity with mental health issues. Anxiety, depression, and schizophrenia have been reported in some individuals with LMS [7]. These findings suggest an underlying connection between the neurodevelopmental aspects of LMS and the occurrence of psychiatric disorders, although further research is needed to confirm this association.

Given the complexity of LMS, particularly when psychiatric symptoms are present, patient management can be challenging. Individualized treatment and personalized follow-up are essential for improving the quality of life for patients. A multidisciplinary approach involving neurology, psychiatry, endocrinology, and other specialties is vital to address both the primary symptoms of LMS and any associated mental health conditions. Continuous monitoring of these rare cases is important, as they are often unfamiliar to clinicians but offer valuable opportunities to refine treatment strategies and improve outcomes for affected individuals [2].

This study aims to contribute to the understanding of psychiatric disorders in the context of LMS by presenting a detailed case analysis of an individual affected by both LMS and a psychotic disorder. A review of the literature revealed no similar case studies published in major databases, including PubMed, underscoring the importance of this case. By examining the clinical presentation, treatment strategies, and outcomes, this study seeks to provide insights that could enhance the management and care of similar patients in the future.

## Case presentation

Patient demographics and background

The patient is a 35-year-old single Saudi male born from a consanguineous marriage. He holds a bachelor's degree in Islamic studies, although his academic journey was marked by significant challenges due to developmental delays and cognitive difficulties. Despite these obstacles, he successfully completed his studies with the support of specialized resources and accommodations. His family consists of his parents, both of whom are teachers and are currently 60 years old, and five siblings, one of whom also has LMS. There is no family history of psychiatric illness, and the patient has no history of drug abuse.

Developmental and medical history

The patient’s developmental history is notable for several delays and difficulties. Born weighing 4 kg after an uncomplicated pregnancy, he exhibited developmental delays, including delayed speech acquisition, with his first word spoken at the age of 4. His developmental progress was markedly different from that of his siblings, with significant challenges in movement, comprehension, and expression. He had difficulty recognizing colors, naming objects, and often exhibited unintelligible speech. Additionally, he experienced poor eyesight, requiring frequent changes in his glasses, and became blind at the age of 33. During his schooling, he struggled academically, repeating the first grade for three years before transferring to a private school with additional support.

The patient has a known medical history of LMS and underwent a laparoscopic cholecystectomy four years ago. On physical examination, his blood pressure was slightly elevated at 133/97 mmHg, and a resting tremor was observed in his right hand. He is 171 cm tall, weighs 150 kg, and has a body mass index (BMI) of 51, indicating obesity. Laboratory tests, listed in Table 1, show elevated levels of total cholesterol, triglycerides, and low-density lipoprotein (LDL) cholesterol, indicating a high risk for atherosclerosis. This is particularly concerning given the presence of additional risk factors such as obesity.

**Table 1:** Laboratory results HDL: high-density lipoprotein, LDL: low-density lipoprotein, HbA1c: glycated hemoglobin.

Test Name	Result	Reference Range
Total cholesterol	6.68 mmol/L	<5.17 mmol/L
HDL cholesterol	1.16 mmol/L	>1.55 mmol/L
LDL cholesterol	4.54 mmol/L	<2.60 mmol/L
Triglyceride	2.15 mmol/L	<1.70 mmol/L
HbA1c	4.9%	4-5.6 %

Psychiatric history and current condition

The onset of the patient’s psychiatric symptoms began at the age of 15, characterized by social isolation and reduced interaction with family and friends. However, his condition deteriorated significantly two years ago, at the age of 33, when he began experiencing distressing auditory hallucinations. He reported hearing voices that spoke about him and his family members, particularly his mother. These voices led to the development of a secondary delusional belief that his mother was involved in a sexual relationship with his cousin and engaged in other sexual activities. This belief heightened his fears and made him unable to sleep alone. The intensity of his hallucinations increased, making him irritable and aggressive toward his mother, and he threatened violent acts toward his cousin, claiming that he had overheard him with his mother. Additionally, the patient experienced tactile hallucinations, feeling as if ants were crawling on his body. Over a period of two months, he neglected his basic needs, including eating, drinking, and performing daily activities.

At the persistent request of his parents, the patient eventually sought medical assistance and was diagnosed with psychosis related to LMS. He was initially prescribed aripiprazole, titrated gradually to 20 mg daily, which led to significant improvement in his symptoms but caused a resting tremor in his right hand. As a result, his dosage was reduced to 10 mg daily, but this reduction triggered a resurgence of his hallucinations. Currently, he is taking aripiprazole 15 mg daily, which has helped stabilize his condition. To alleviate the negative symptoms associated with his psychotic disorder, a trial of escitalopram 10 mg was conducted, but it did not result in noticeable improvement and was subsequently discontinued.

Mental state examination

Upon mental status examination, the patient appeared appropriate for his stated age. He was cooperative, though he exhibited poor eye contact, attributed to his blindness. There were no signs of psychomotor agitation or retardation. His speech was normal in rate, rhythm, and volume. He described his mood as "good," though his affect was restricted. His thought process was coherent and goal-directed, with no active suicidal or homicidal ideation. The patient exhibited occasional delusions of jealousy, manifested as baseless accusations of infidelity against his mother and excessive monitoring of her actions. No other significant perceptual abnormalities were noted. Formal cognitive testing was not completed, as the patient was uncooperative during the assessment. However, his overall presentation suggested possible cognitive impairment, reflected in difficulties with attention, processing speed, and executive functioning, as well as his history of developmental delays and learning disabilities. In summary, this patient presents with a complex case involving LMS and secondary psychosis, manifesting both physical and psychiatric challenges. His treatment plan is being adjusted to optimize symptom control while minimizing side effects. Continuous monitoring and multidisciplinary support are essential for managing his condition effectively.

## Discussion

LMS is a rare autosomal recessive genetic disorder characterized by a combination of neurological, visual, and metabolic abnormalities [1]. The coexistence of LMS with psychiatric disorders, such as psychosis, further complicates the clinical picture and necessitates a nuanced approach to treatment. This case exemplifies the relationship between psychiatric disorders and LMS.

The patient’s psychiatric symptoms, that is, hallucinations, delusions, and severe behavioral disturbances, highlight the complex interplay between LMS and psychotic disorders [8]. Hallucinations, particularly auditory ones, are well-documented in various psychiatric conditions, but their emergence in the context of LMS presents unique challenges [9]. The patient’s belief that his mother engaged in inappropriate activities, along with tactile hallucinations of ants crawling on his body, underscores the need for comprehensive psychiatric evaluation. This evaluation would involve a detailed clinical assessment, standardized psychometric testing, and a multidisciplinary approach, including input from neurologists and psychologists. Monitoring the effects of medications and involving the patient’s family in treatment planning is also essential. Regular follow-up visits would ensure continuous monitoring of symptoms and allow for timely adjustments to the care plan.

The impact of psychotic symptoms on the patient’s behavior was significant, leading to aggression and neglect of self-care. This behavioral deterioration aligns with findings that psychotic disorders can exacerbate maladaptive behaviors and complicate self-care routines in patients with underlying neurodevelopmental disorders [10,11]. The patient’s aggressive behavior primarily manifested as verbal outbursts and threatening gestures directed toward his family, particularly his mother. He frequently expressed anger and frustration through accusatory statements and threats of harm toward family members, including his cousin, whom he believed was involved in inappropriate activities with his mother. This aggression created an environment of fear and distress, causing family members to feel unsafe and anxious, which further exacerbated the patient’s social isolation and led to his neglect of basic needs. Such behavior reflects severe distress and impairment, which is critical to address in treatment planning. The absence of a family history of psychiatric illness, combined with the patient’s specific tactile hallucinations and delusional beliefs related to his condition, strongly suggests that the psychotic symptoms are secondary to LMS.

Treatment with antipsychotic medications, specifically aripiprazole, was initially beneficial but led to the development of a resting tremor, a known side effect of such medications [12]. Several metabolically sparing agents were suggested, including brexpiprazole, cariprazine, ziprasidone, and aripiprazole. Aripiprazole was chosen due to its metabolic-sparing properties, which were essential given the patient’s baseline obesity, as well as the availability of a depot injection for long-term management in case of poor adherence. This highlights the challenge of balancing efficacy and tolerability in pharmacological treatment for patients with comorbid conditions. The adjustment of aripiprazole dosage in response to side effects illustrates the difficulties in managing the patient’s psychiatric symptoms-such as hallucinations and delusions-which are secondary to LMS. The return of psychotic symptoms following the dosage reduction emphasizes the importance of balancing effective antipsychotic treatment with managing primary LMS symptoms, including obesity and cognitive impairments [2].

A trial of escitalopram, a selective serotonin reuptake inhibitor (SSRI) aimed at improving the negative symptoms associated with psychosis, was discontinued due to a lack of improvement and the patient’s non-compliance with multiple medications. This underscores the importance of ensuring high patient adherence to medication regimens. SSRIs are often used alongside antipsychotics to manage mood and depressive symptoms; however, their effectiveness can be variable, especially in patients with complex presentations [13].

The patient’s elevated cholesterol levels and obesity add another layer of complexity. These comorbid conditions significantly impact overall health and may result from both the underlying syndrome and medication side effects [14]. Managing these metabolic disturbances through dietary modifications, exercise, and potentially medications such as statins can improve overall health, reduce the risk of long-term complications such as atherosclerosis, and enhance the patient’s quality of life. In patients with LMS, where metabolic abnormalities are part of the syndrome's presentation, a comprehensive approach addressing both psychiatric and physical health is essential for achieving better long-term outcomes.

One limitation of this case report is the absence of genetic and hematological testing, which could have provided more precise information about the specific mutations responsible for LMS. Identifying these genetic mutations could offer insights into the underlying mechanisms of both physical and psychiatric symptoms, potentially guiding treatment decisions. Therefore, genetic testing is recommended to identify relevant mutations and support personalized care. Additionally, non-invasive prenatal testing (NIPT) should be considered for the patient’s siblings during future pregnancies after marriage, as this can help identify potential genetic risks in offspring. Incorporating such genetic evaluations into routine management would not only enhance the understanding of the disease but also provide valuable preventive measures for families affected by LMS.

## Conclusions

This case study enhances our understanding of the manifestations and management of psychosis in patients with LMS. A multidisciplinary treatment approach, involving psychiatry, neurology, endocrinology, and metabolic care, is essential to ensure comprehensive management of both the psychiatric and physical health aspects of the syndrome. Given the genetic nature of LMS, we recommend genetic testing and counseling for the patient’s family, especially during family planning. NIPT can be offered to siblings after marriage to identify any genetic abnormalities early in pregnancies.

Further research is needed to develop targeted treatment strategies that address the unique needs of patients with LMS and comorbid psychiatric disorders. Studies focusing on the long-term outcomes of specific psychotropic medications, as well as the roles of genetic and environmental factors in contributing to psychiatric symptoms in LMS, would provide valuable insights. Understanding these factors could lead to more effective treatment regimens, improve patient adherence, and enhance the quality of life for individuals affected by LMS.

## References

[1] Kumar A, Husain A Sr, Saleem A, Khawaja UA, Virani S (2020). Laurence-Moon-Bardet-Biedl syndrome: a rare case with a literature review. Cureus.

[2] Khan OA, Majeed R, Saad M, Khan A, Ghassan A (2019). Rarity of Laurence Moon Bardet Biedl syndrome and its poor management in the Pakistani population. Cureus.

[3] Kaleem S, Srirangadhamu Gopu S, Ishfaq L, Afroze S, Parvez M, Mulaka GS, Venugopal V (2023). Laurence-Moon-Bardet Biedl syndrome with cholelithiasis. Cureus.

[4] Dave M, Goyal A, Saini RG, Dave G (2023). Laurence-Moon-Bardet-Biedl syndrome: a rare case report. Indian J Clin Pract.

[5] Munib N, Haider I, Malik SE (2022). Laurence-Moon-Bardet-Biedl syndrome: a case report. J Med Sci.

[6] McGrath JJ, Lim CC, Plana-Ripoll O (2020). Comorbidity within mental disorders: a comprehensive analysis based on 145 990 survey respondents from 27 countries. Epidemiol Psychiatr Sci.

[7] Qadar LT, Ahmed ZM, Munawar M, Hasan CA, Iqbal SU (2019). Laurence-Moon-Bardet-Biedl syndrome with coexisting abdominal distension and positive fluid thrill: a rare manifestation reported in Karachi, Pakistan. Cureus.

[8] Weiss M, Meshulam B, Wijsenbeek H (1981). The possible relationship between Laurence-Moon-Biedl-Bardet syndrome and a schizophrenic-like psychosis. J Nerv Ment Dis.

[9] Kumar S, Soren S, Chaudhury S (2009). Hallucinations: etiology and clinical implications. Ind Psychiatry J.

[10] Gkintoni E, Skokou M, Gourzis P (2024). Integrating clinical neuropsychology and psychotic spectrum disorders: a systematic analysis of cognitive dynamics, interventions, and underlying mechanisms. Medicina (Kaunas).

[11] Maj M, van Os J, De Hert M (2021). The clinical characterization of the patient with primary psychosis aimed at personalization of management. World Psychiatry.

[12] Di Sciascio G, Riva MA (2015). Aripiprazole: from pharmacological profile to clinical use. Neuropsychiatr Dis Treat.

[13] Stroup TS, Gray N (2018). Management of common adverse effects of antipsychotic medications. World Psychiatry.

[14] Mc Auley MT (2020). Effects of obesity on cholesterol metabolism and its implications for healthy ageing. Nutr Res Rev.

